# The irreplaceable role of pathology for the clinical translation of patient-derived organoids in precision medicine

**DOI:** 10.1093/pcmedi/pbaf032

**Published:** 2025-11-14

**Authors:** Zuoyu Liang, Ping Yang, Xinglong Zhu, Yihong Liu, Mumin Shao, Zaiyu Yang, Ji Bao

**Affiliations:** Department of Pathology, Institute of Clinical Pathology, Key Laboratory of Transplant Engineering and Immunology, West China Hospital, Sichuan University, Chengdu 610041, China; Department of Pathology, Institute of Clinical Pathology, Key Laboratory of Transplant Engineering and Immunology, West China Hospital, Sichuan University, Chengdu 610041, China; Department of Pathology, Institute of Clinical Pathology, Key Laboratory of Transplant Engineering and Immunology, West China Hospital, Sichuan University, Chengdu 610041, China; Center for Information and Language Processing, University of Munich, Munich 80539, Germany; Department of Pathology, Shenzhen Traditional Chinese Medicine Hospital, Shenzhen 518033, China; Department of Pathology, Fourth Clinical Medical College of Guangzhou University of Traditional Chinese Medicine, Shenzhen 518033, China; Biology Department, Georgia State University, Atlanta, GA 30302, United States; Department of Pathology, Institute of Clinical Pathology, Key Laboratory of Transplant Engineering and Immunology, West China Hospital, Sichuan University, Chengdu 610041, China; State Key Laboratory of Kidney Diseases, Chinese PLA General Hospital, Beijing 100004, China

Dear Editor,

Patient-derived organoids (PDOs) are directly established via *in vitro* culture of patients’ tumor samples, with the capacity to highly recapitulate the physiological traits, pathological states, and biological behaviors of the original tumor tissues. As an optimal bridge connecting *in vitro* and *in vivo* models, PDOs have been successfully constructed from tumor tissues of multiple organs, including the brain, breast, kidney, liver, lung, pancreas, and small intestine, and are currently applied across a broad spectrum of fields such as basic research, tumor model construction, drug development, biobank establishment, and personalized therapy [1]. Their superior ability to mimic the structural and functional features of tumor tissues *in vitro* stems from their retention of cell–cell interactions, cell–extracellular matrix crosstalk, and pathophysiological characteristics analogous to those of native tumors. Critically, PDOs retain intratumoral heterogeneity, a key attribute that enables antitumor drug screening and clinical treatment guidance, thereby demonstrating immense potential in precision medicine [2]. Notably, the approval of the FDA Modernization Act 2.0 in December 2022 eliminated the mandatory requirement for animal testing in new drug development and encouraged the gradual adoption of alternative models where feasible. Furthermore, on 10 April 2025, the FDA announced a draft plan to phase out animal use in the development of monoclonal antibodies and other pharmaceutical products. These regulatory advancements undoubtedly serve as a significant recognition of PDOs’ value in drug development and precision therapy, positioning PDOs as promising alternatives to animal experiments.

**Figure 1.**
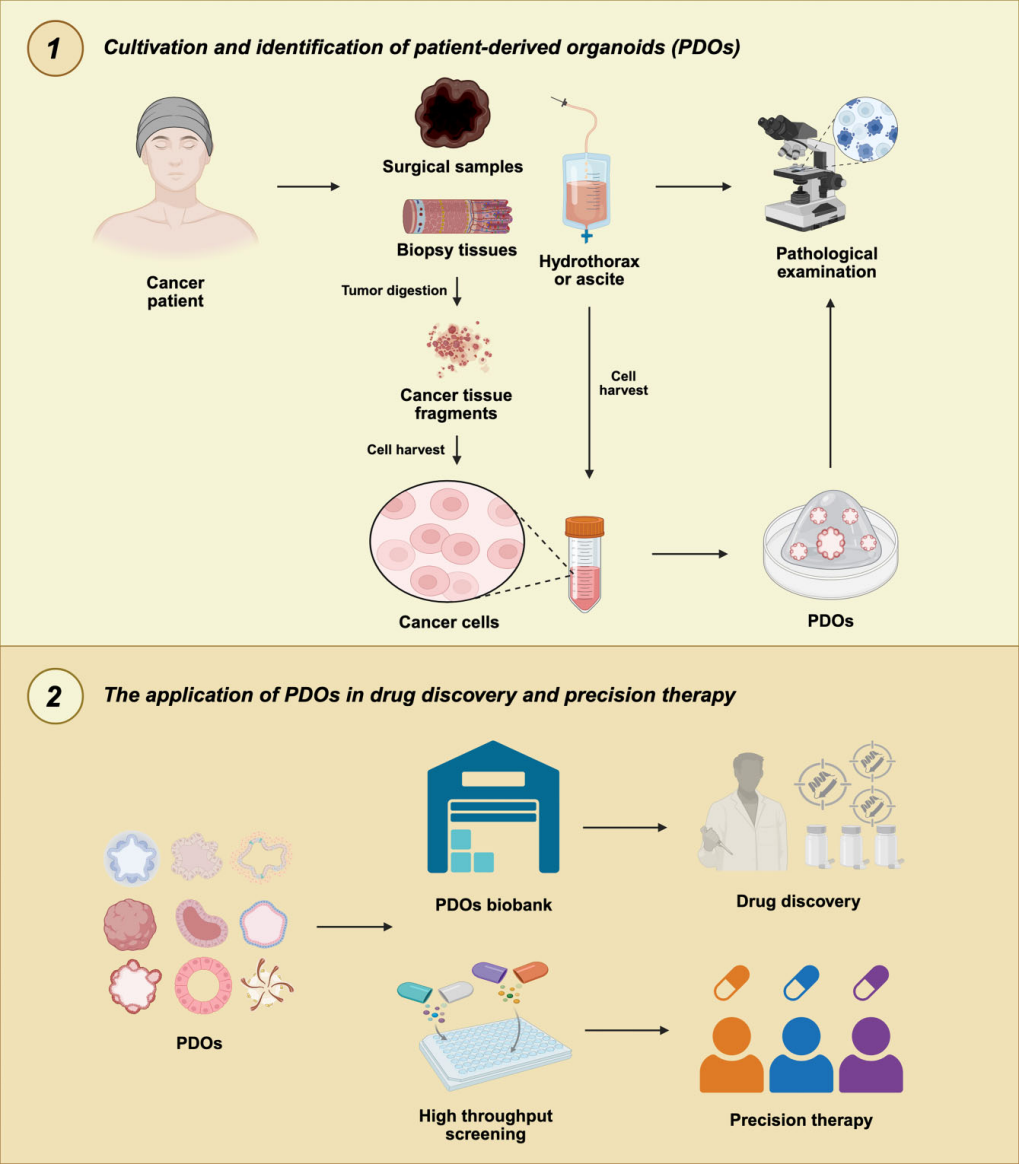
Schematic overview of cultivation and application of PDOs in precision therapy.

Pathological examination is the cornerstone of PDO research and remains indispensable throughout the workflow (Fig. 1). Microscopic examination identifies highly viable areas and excludes necrotic regions, which is critical for the successful establishment of PDOs [3]. Following establishment, the reliability of PDOs must be verified by morphological examination, immunohistochemistry, and molecular profiling to ensure faithful representation of the original tumor. This is a prerequisite for guiding therapeutic decisions. Without such validation, the risk of genotype–phenotype mismatch increases, undermining the models’ predictive accuracy. For instance, breast-cancer PDOs that show genomic HER2 amplification yet fail to express detectable HER2 protein are unreliable for predicting the efficacy of HER2-targeted therapies. Moreover, in interpreting drug sensitivity data, the correlation among pathological morphology, molecular signatures, and clinical outcomes provides essential biological context and clinical relevance [2]. Pathological validation is also crucial for the emerging "digital organoids"—*in silico* models built from validated PDO multi-omics data—since the accuracy of their virtual predictions depends on the pathological and biological fidelity of the parent PDOs [4]. Beyond validating PDOs themselves, these models also hold potential to advance pathological diagnosis. For example, PDOs can expand small, precious biopsy specimens into a renewable “living biobank” that enables deep phenotyping of rare tumors, thereby supporting the refinement of diagnostic criteria. As a case in point, such PDOs can be established from fine-needle aspiration samples of lung and head-and-neck cancers with a success rate >80%, thereby offering an alternative diagnostic option for patients who are unsuitable for surgical biopsy [3]. As cutting-edge technologies advance rapidly, PDO models are expected to become an important complementary tool for pathological diagnosis. Integrating PDO workflows into routine pathology promises to transform cancer care from empirical approaches to personalized therapeutic regimens guided by *in vitro* tumor response. Realizing this vision requires continuous innovation in model complexity, standardization of protocols, and large-scale prospective clinical validation.

Nevertheless, despite their significant promise in precision medicine, PDOs still face challenges in model integrity and pathological applicability. First, PDOs have difficulty in fully recapitulating the cellular composition and intercellular crosstalk of the primary tumor microenvironment (TME), resulting in insufficient simulation of stromal invasion, angiogenesis, and immune infiltration. Second, long-term culture selectively enriches tumor subclones adapted to *in vitro* conditions, driving phenotypic drift away from the parental tumor. At the pathological level, the slow expansion kinetics of PDOs often limits the acquisition of sufficient samples for paraffin embedding. Furthermore, obstacles including prolonged establishment timelines, inconsistent success rates, and the absence of standardized protocols encompassing culture, morphological assessment, and molecular validation collectively hinder the pathological interpretation and clinical translation of PDOs.

Future development of PDOs will be propelled by the integration of cutting-edge analytical technologies, particularly in 3D pathology and artificial intelligence (AI). The primary goal is to reconstruct the tumor TME by incorporating stromal and immune cells to develop more physiologically relevant “immune organoids” or “multicellular organoids” that better recapitulate *in vivo* responses to immunotherapy and targeted therapy. A key enabling technology is the recent development of 3D pathology, which has brought revolutionary advances to PDO analysis [5]. Stimulated Raman scattering (SRS) microscopy enables label-free, non-invasive 3D imaging of live PDOs while preserving their viability for longitudinal studies [6]. The virtual hematoxylin–eosin-stained images generated by SRS can provide detailed histological information, allowing researchers to conduct long-term dynamic observations of the same organoid. The optical sectioning capability of SRS enables reconstruction of the 3D architecture of organoids, revealing subcellular structural details, thus offering valuable insights into tumor heterogeneity and therapy response. For instance, researchers at Tongji Hospital (Wuhan, China) have developed a 450 nm ultra-thin non-diffracting light-sheet microscope that performs virtual sectioning of cleared tissues. By overcoming 2D imaging limitations, this technology captures both global and subcellular details of specimens, facilitating visualization of 3D vascular networks and immune infiltration, and is now in clinical trials for the translational application of 3D pathology in clinical diagnosis. Furthermore, AI and deep learning, particularly convolutional neural networks, will fundamentally transform PDO analysis [7]. The automation of morphological feature recognition and classification in high-throughput images, as well as prediction of drug responses from bright-field images, can drastically improve the efficiency and objectivity of clinical decision-making. AI-driven segmentation algorithms can automatically identify and quantify different cell populations, detect therapeutic responses, and accurately predict treatment efficacy based on PDO features, facilitating large-scale drug screening [8]. Moreover, AI models trained on pathological features of PDOs (e.g. cellular arrangements, nuclear-to-cytoplasmic ratios) and molecular data can assist pathologists in rapidly determining tumor subtypes, key molecular events, and therapeutic sensitivity, thereby reducing inter-observer disagreement in PDO-based morphological classification. The integration of these technologies with PDOs is fostering a new, highly quantitative approach to organoid analysis.

Propelled by these technological advancements, the core direction for PDO development lies in their deep integration throughout the entire cancer-care continuum, transforming them into dynamic decision-support systems that seamlessly connect diagnostics and therapy: specifically, in drug resistance research, PDOs enable the dynamic tracking of genotypic and phenotypic evolution in tumor clones escaping drug pressure, guiding the design of effective combination therapies to circumvent resistance; in drug development, large-scale PDO biobanks serve as preclinical avatars for evaluating drug efficacy across diverse genetic backgrounds; and in clinical translation, these living biobanks provide expandable repositories for precious patient specimens, thereby facilitating research on rare tumors. Furthermore, integrating PDOs with liquid biopsy and multi-omics sequencing establishes a dynamic monitoring framework for tracking resistance mutations and phenotypic shifts throughout a patient's treatment course. Complemented by 3D virtual pathology for spatial analysis and AI for image-based prediction, the synergy between PDOs and emerging technologies like organ-on-a-chip (which uses microfluidics to simulate physiological flow) will continually broaden their precision medicine applications.

In summary, PDOs are reshaping precision oncology by serving as "patient tumor avatars", transforming drug testing from an exploratory practice into a core component of standardized care (Fig. 1). For example, glioblastoma PDOs have accurately predicted patient responses to chimeric antigen receptor modified T cell (CAR-T) therapy and identified post-treatment clonal evolution [9]. With progress in automated bioprinting and microfluidics, the timeline from biopsy to functional PDO arrays can be shortened to days, creating a practical window for guiding first-line therapy [8]. This evolution signifies a shift from experience-based to data-driven oncology, elevating pathology from traditional morphology to an integrated discipline. Pathology remains central to this transition by ensuring accuracy throughout PDO establishment, validation, and interpretation—thus safeguarding the biological relevance and clinical utility of organoid-based assays. Supported by PDO-integrated platforms, pathologists will increasingly contribute to therapeutic planning, transitioning from diagnosticians to key decision-making partners. Thus, PDOs critically bridge the gap between bench and bedside, with the ultimate goal of enhancing patient survival and quality of life.
